# Virtual Reality Relaxation for Reducing Perceived Stress of Intensive Care Nurses During the COVID-19 Pandemic

**DOI:** 10.3389/fpsyg.2021.706527

**Published:** 2021-09-29

**Authors:** J. W. H. Mathijs Nijland, Wim Veling, Bart P. Lestestuiver, Catheleine M. G. Van Driel

**Affiliations:** ^1^ Department of Psychiatry, University Medical Centre Groningen, Groningen, Netherlands; ^2^ VRelax B.V., Groningen, Netherlands

**Keywords:** stress, relaxation, virtual reality, COVID-19, ICU nurses

## Abstract

During the COVID-19 pandemic ICU nurses endure high levels of stress. VR relaxation (VRelax, containing 360° immersive environments) provides an easy-to-use and effective means to induce positive affect and reduce perceived stress. We investigated feasibility and immediate effect on perceived stress of VRelax use by ICU nurses during work shifts. ICU nurses working with COVID-19 patients in an academic hospital could use VRelax as a 10-min break during their shift. Primary outcome was the difference between perceived stress immediately before and after VRelax use measured by a single-question VAS-stress scale. Statistically significant difference of the mean VAS-stress before and after use was determined using the paired *t* student test. A socio-demographic questionnaire, a questionnaire on perceived stress and stress resilience and VRelax user experiences were sent by email. Eighty-six (26%) nurses used VRelax at least once; 77% (*N*=66) of these filled out the VAS-stress scale before and after use of VRelax. Mean perceived stress lowered with 39.9% after use of VRelax (mean difference=14.0, SD=13.3, *p*<0.005). Mean score on the perceived stress scale-10 was 11.4 (SD=6.50), mean score on the Connor-Davidson Resilience Scale-10 was 29.0 (SD=5.51). Sixty-two percentage of the ICU nurses thought VRelax was helpful to reduce stress. Main barrier for use was a high workload. It is feasible for nurses to use VRelax in an ICU context. VRelax is an effective intervention to reduce immediate perceived stress and is of added value in stressful situations as during the COVID-19 pandemic, inducing a positive affective state and lowering perceived stress.

## Introduction

Long-term exposure to high levels of stress is detrimental to the mental health and sustainable employability of healthcare professionals (Dewa et al., 2014). When individuals experience stress, they appraise a specific challenge as potentially exceeding their coping strategies (Folkman, 2010). When this stressful situation endures, it can lead to high emotional burden, fatigue, and a feeling of loss of control (Harris, 2001).

During the first wave of COVID-19 infections in the spring of 2020, healthcare professionals reported significantly more stress than before the pandemic (Erquicia et al., 2020; Vizheh et al., 2020). In 2020, substantial rates of probable mental health disorders were reported among ICU professionals, especially in nurses (Greenberg et al., 2021). More in general it is stated that healthcare professionals experienced significant levels of anxiety, depression, insomnia, and distress during the COVID-19 pandemic, especially nurses, working in close contact with COVID-19 are at risk (De Kock et al., 2021; Wu et al., 2021). Recent research suggests raised levels of burnout when screened on burnout symptoms among healthcare professionals during the COVID-19 pandemic (Dobson et al., 2021). The long-term effects on healthcare professionals are still unknown. However, 2years after the 2003 SARS outbreak, staff directly involved in care for SARS patients reported 10 percent more burnout symptoms and almost 15 percent more psychological distress than healthcare professionals not exposed to the care for SARS patients (Maunder et al., 2006). In addition, chronic stress is associated with impaired cognitive functioning and mental disorders such as anxiety and depression (Eisler and Polak, 1971; Weber et al., 2009). This makes the expected burden on the individual healthcare professionals (e.g., mental health complaints and decreased job performance) and the burden on society (e.g., personnel shortages, overwhelmed healthcare systems, and additional healthcare costs) substantial (West et al., 2018). This signifies the need to improve the effects of stress associated with the COVID-19 pandemic and future outbreaks on healthcare workers.

A starting point to ameliorate these effects is enhancing individuals’ coping strategies to increase their stress resilience. Emotion-based coping (i.e., managing own emotions) and problem-focus coping (i.e., managing the problem) are usually the primary coping strategies to be employed (Folkman and Greer, 2000). When these strategies lead to the resolve of the stressor the coping effort ceases. However, in the case of the COVID-19 pandemic the stressor did not resolve, but endured over a longer period of time. This uncontrollable and persistent situation leads to another coping strategy being activated, namely meaning-focused coping. Meaning-focused coping is aimed at maintaining well-being under an enduring stressful situation by relinquishing unattainable goals (e.g., immediate resolve of COVID-19 pandemic) and formulating new ones (e.g., maintain (mental) well-being despite the pandemic). Meaning-focused coping often increases positive affect, providing psychological “time out” from the distress motivating sustained coping efforts (Folkman, 2010).

VR relaxation can support emotion-based coping, as well as meaning-focused coping strategies by leading to a positive affective state and immediate stress reduction (Veling et al., 2021). VR relaxation is easy to use and has an advantage over regular relaxation exercises as it requires far less effort in terms of concentration and attention and leads to a larger increase in positive affect (e.g., happiness, etc.; Veling et al., 2021). Our VR relaxation intervention (VRelax), consisting of calming natural environments, has shown to lead to immediate decrease in perceived stress and an improvement in overall positive affective states in people with psychiatric problems (Veling et al., 2021).

We aimed to investigate whether use of VRelax by ICU nurses working with COVID-19 patients would be feasible during ICU work shifts. Therefore, we collected information on experience with the use of VRelax, focusing on known factors for the acceptance of new technology, perceived usefulness, and perceived ease of use, as is described in the technology acceptance model (Davis, 1989). Furthermore, we investigated whether using VRelax during 10-min breaks would reduce experienced stress immediately.

## Participants and Methods

### Study Design

This pre–post-intervention study with assessments immediately before and after VR relaxation sessions was set up in May 2020, during the first wave of the COVID-19 pandemic in the Netherlands. Due to the acute and challenging situation and the aim of the study to provide VR relaxation for staff during work shifts on the COVID-19 wards, pre–post-assessments were restricted to a single question. A control group was not included as we aimed to provide the expected benefit of VRelax to as many ICU nurses as possible. The Medical Ethical Committee of the University Medical Centre Groningen (UMCG) declared in May 2020 that the Medical Research Involving Human Subjects Act did not apply to this study. It was conducted in accordance with the relevant Dutch legislation (e.g., The Data Protection Act) and the UMCG Research Code, version May 2019. Data of the study were collected and managed using REDcap hosted at the UMCG (Harris et al., 2009, 2019).

### Participants and Procedure

All 326 ICU nurses of the UMCG working on one of the four intensive care units for COVID-19 patients were invited to use VRelax during their shifts. No specific exclusion criteria were given, other than having a history of photosensitive epilepsy. From May until June 2020, ICU nurses had access to VRelax. The nurses were encouraged by trained (para)medical students or by their team leader to use VRelax as a short break during their shift. On all four ICUs, a separate room was available for VRelax, which was equipped with a stand-alone head-mounted device (Oculus Go) running the VRelax application and a comfortable swivel armchair. Assistance from the trained (para)medical students during use consisted of explanation and demonstration of how to use VRelax. Furthermore, before and after the use of VRelax the students collected VAS-stress scores. During the evening and night VRelax was available without assistance and ICU nurses could fill out the before and after VAS-stress score themselves. In June, after the pre–post-phase of the study, all ICU nurses, regardless of VRelax use, received a questionnaire by email (see measurements and assessments).

### Hygiene

After consultation with the hospital hygiene and infection prevention department, specific hygiene precautions were taken. Besides general hygiene precautions (e.g., hand hygiene), users wore surgical caps during use and before and after every use all the parts of the device that came into contact with the user were disinfected with hydrogen peroxide wipes (ECOLAB Indicin® OxyWipe and Bacillol 30).

### Intervention

**Image 1:**
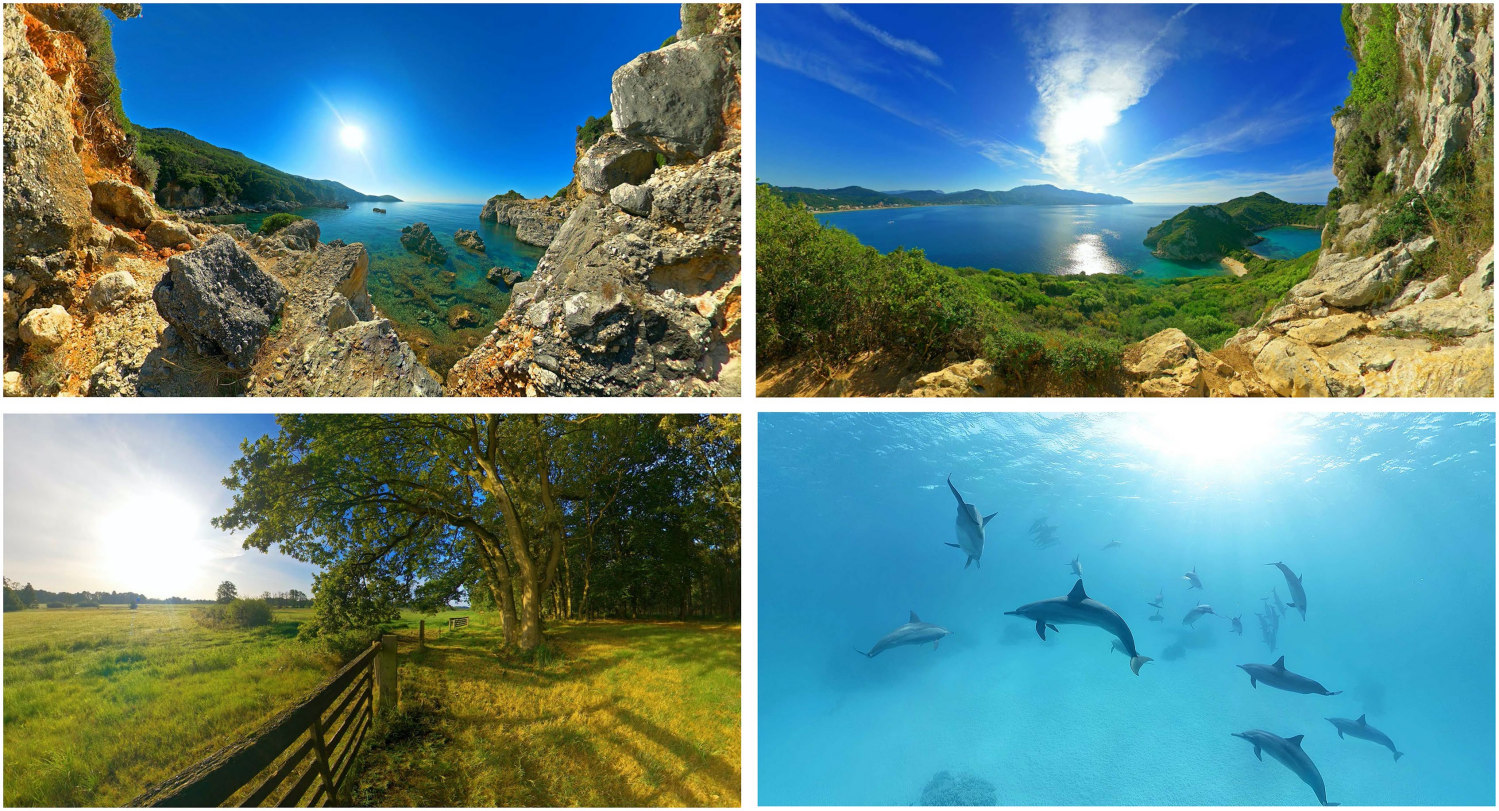
VRelax environments. Source: VRelax BV.

The device was an Oculus Go stand-alone head mounted display running the VRelax application. VRelax is an easy-to-use, plug-and-play application. The participants could navigate through high-quality immersive 360-degree videos of calming natural environments (see Image 1). Options include walking on a beach and underwater swimming with wild dolphins. The videos were inclusive embedded interactive elements (e.g., a game of popping underwater air bubbles, shooting start in a night sky, and audio tracks of relaxation exercises). The recommended minimal time of use was 10min, a longer duration was allowed. Actual use duration was not collected. The participants were also not limited in the frequency of use of the device.

### Measurements and Assessments

The following socio-demographic characteristics were obtained: age, sex, and mean years ICU work experience. The primary outcome of interest was the level of perceived stress immediate before and after VRelax. The participants were asked “How much stress do you perceive at this moment?” Their answer was rated on a visual analogue scale-stress (VAS-stress) ranging from 0 to 100, immediately before and after using VRelax. Furthermore, participants indicated if this was their first-time use or repeated use to avoid double data entries.

After the intervention period all ICU nurses received a digital questionnaire. For VRelax users, specific questions (closed-ended questions and an open-ended question) about usage were included (e.g., How often did you use the VRelax-application? How did you experience using VRelax? Did you find use VRelax helpful?). Regardless of VRelax use, all ICU nurses were invited to fill out a questionnaire on perceived stress (Perceived Stress Scale; Cohen et al., 1983) and mental resilience (Connor-Davidson Resilience Scale; Campbell-Sills and Stein, 2007; Davidson, 2020, unpublished).

The 10-item perceived stress scale (PSS-10) is a self-reported questionnaire that measures experienced stressful moments during the last month. Ten questions with a five-point scale (ranging from 0 till 4) are used (0 for statements that never occurred, 4 for statements that occurred very often). The scores to the four positively stated items (items 4, 5, 7, and 8) are reversed (e.g., 0=4, 1=3, and 4=0). With a maximum score of 40, a score of 12.1 for men and 13.7 for woman was considered as average as described in their norm table (Cohen et al., 1983). In terms of internal consistency, test–retest reliability and validity does the PSS-10 met the criteria, suggesting a questionnaire with established acceptable psychometrics (Lee, 2012).

The 10-item Connor-Davidson Resilience Scale (CD-RISC-10) is a self-reported questionnaire and measures degree of (stress) resilience. The CD-RISC-10 was developed with posttraumatic stress disorder as starting point, but is validated in different population types, including general population. Respondents rate on statements on a five-point scale from 0 (not true at all) to 4 (true nearly all the time), with a maximum of 40. The questions are divided into five domains (e.g., flexibility, self-efficacy, ability to regulate emotion, optimism, and cognitive focus/maintaining attention under stress; Davidson, 2020, unpublished).

Scores on the CD-RISC-10 are divided into quartiles: 25th %=29; 50th %=32; and 75th %=36. A score in the lowest quartile suggest low resilience, a score in the highest quartile indicates high resilience (Davidson, 2020, unpublished). Mean score of 31.8 (SD=5.4) was obtained on the CD-RISC-10 based on a community sample in the United States (Campbell-Sills et al., 2009).

The CD-RISC-10 is a unidimensional scale with good psychometric properties, which encloses good internal consistency moderate test–retest reliability and good construct validity (Windle et al., 2011; Tourunen et al., 2021).

### Data Analysis

Socio-demographic characteristics were described using frequency, percentage, or if better suited mean or median and SD. Users and nonusers of VRelax were compared to investigate whether there was a statistically significant difference in terms of age, ICU experience and scores on the PSS-10 and CD-RISC-10, using the independent samples *T* test. The Chi-square test was used to investigate whether gender and use of VRelax were statistically significantly related. The independent samples *T* test was used to identify whether there was a relation between gender, age (older or younger than 40years), and years of experience (more or less than 10years) with respect to the mean scores of the PSS-10 and CD-RISC-10. Mean VAS-stress before VRelax and mean VAS-stress after VRelax were compared using the paired t student test. For each case we calculated a Reliable Change Index (RCI) as described by Jacobson and Truax (1991) and indicates whether a change in a person reflects “real” improvement or is possibly due to measurement error. An outcome <−1.96 was considered a reliable change. A test–retest value for the VAS-stress was not available; therefore, the most conservative test–retest value for VAS-anxiety was used (*r*=0.44; Abend et al., 2014). For the calculation of the standard error of measurement (S_E_) we used the SD of the pretreatment group (=25.72). Afterward, percentage of cases with a score <−1.96 on the RCI was calculated. Only pre–post-scores during first-time use of VRelax were included in the analyses to avoid including pre–post-data of the same subject multiple times and avoid heterogeneity of effect due to differences between first-time use and repeated use. All value of *p* were two-tailed and considered statistically significant if *p*<0.05. We used SPSS 23 (IBM Corp., Armonk, NY, United States) for all quantitative analyses.

To gain understanding of the experiences with using VRelax, participants filled out a structured questionnaire on ease of use, effect, and availability of VRelax (at home or on the ward). Furthermore, they answered an open question on user experience namely “How did you experience using VRelax?.” Comments given on the open question were encoded using the six-step method as recommended for qualitative thematic analysis (Braun and Clarke, 2006). Two authors (JN and BL) analyzed the data independently. After familiarizing with the data, general codes were generated. After searching for themes, the themes were reviewed by JN, BL, and CD, after which the themes were defined and named. Microsoft Word was used for the analysis.

## Results

### Population Description

Twenty-six percent (*N*=86) of all ICU nurses used VRelax at least once in the study period. Seventy-seven percent (*N*=66) of them used VRelax for the first time and filled out the VAS scoring perceived stress before and after use of VRelax. The majority were women (73 of 86, 85%) and on average the participants had 13.2years (SD=10.9) of ICU experience (Table 1). Eighty VRelax users filled out the 10-item Perceived Stress Scale, 75 VRelax users filled out the 10-item Connor-Davidson Resilience Scale. Mean total score on the PSS-10 was 11.4 (SD=6.5), which is below norms of general population and indicates low perceived stress. Mean total score on the CD-RISC-10 was 29.0 (SD=5.5), with a median of 29 which is the cutoff score between the lowest two quartiles, indicating low stress resilience. In comparison, VRelax users did not statistically significantly differ from the non-VRelax users in socio-demographics, ICU experience, and scores on the PSS-10 and CD-RISC-10. See Table 1 for all (sub-)scores. Mean scores on the PSS-10 and CD-RISC-10 did not differ significantly for gender, age (<40 vs. >40), or years of ICU experience (<10 vs. >10), see Table 2. A negative correlation was found between the scores on PSS-10 and CD-RISC-10 with *r*=−0.656 (*p*<0.001), meaning higher levels of perceived stress were correlated with lower stress resilience.

**Table 1:** Descriptive statistics of ICU nurses, VRelax users, and non-VRelax users.

	VRelax users	Non-VRelax users	*p* (*t*)
N (% of total respondents)	86 (62%)	52 (38%)	
Age, years (M, SD)	42.4 (12.1)	41.7 (11.5)	0.767 (0.297)
Gender (N, %)			
Male	13 (15%)	9 (17%)	*X*^2^ (1,138)=0.116, *p* =0.733
Female	73 (85%)	43 (83%)	
Years of ICU experience (M, SD)	13.2 (10.9)	13.5 (10.2)	0.878 (−0.154)
Perceived stress scale-10			
Total score (M, SD)	11.4 (6.50)	11.8 (6.52)	0.776 (−0.285)
CD-Resilience Scale-10			
Flexibility (M, SD)	6.0 (1.38)	6.1 (1.14)	
Self-efficacy (M, SD)	8.8 (1.69)	8.7 (1.47)	
Regulate emotion (M, SD)	2.8 (0.79)	2.9 (0.71)	
Optimism (M, SD)	8.5 (1.97)	8.7 (1.60)	
Maintaining attention under stress (M, SD)	2.9 (0.59)	2.9 (0.70)	
Total score CD-RISC-10 (M, SD)	29.0 (5.51)	29.4 (4.80)	0.697 (−0.391)
Total score CD-RISC-10 (median)	29	29	

**Table 2:** Mean scores on PSS-10 and CD-RISC-10 compared with socio-demographics.

	Perceived stress scale-10			CD-Resilience scale-10		
	*N*	Mean	*p* (*t*)	*N*	Mean	*p* (*t*)
Male	21	10.2	0.312 (−1.016)	20	31.0	0.076 (1.792)
Female	103	11.8		97	28.8	
Age<40years	62	12.1	0.378 (−0.885)	57	28.7	0.403 (0.839)
Age>40years	62	11.0		60	29.5	
<10y work experience	55	11.5	0.736 (0.338)	38	29.3	0.683 (−0.410)
>10y work experience	62	11.9		72	28.2	

### Immediate Effect of VRelax on Perceived Stress

**Figure 1:**
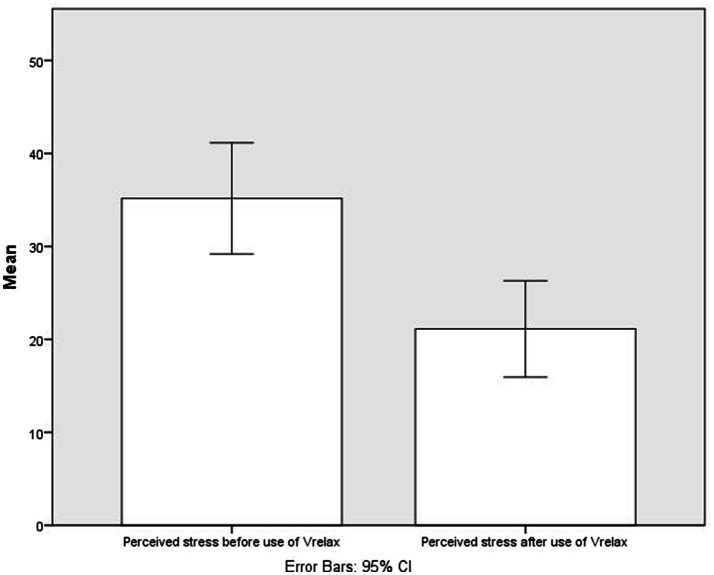
Perceived stress before and after use of VRelax.

After VRelax sessions, the mean perceived stress level was 39.9% lower compared to the level before VRelax sessions. Mean VAS-stress before VRelax was 35.2 (SD=24.3), mean VAS-stress after VRelax was 21.1 (SD=21.1). The mean difference was 14.0 points, SD=13.3; *t*-test value=8.6; *p*<0.005; 95% CI=10.8–17.3 (see Figure 1).

The Reliable Change Index (RCI) was calculated for each case, one case (1.5%) had a score <−1.96.

### Participants’ Comments on Using VRelax

The majority of nurses indicated that stress resilience was important to very important in their work (134 of the 138, 97.1%). Of the nurses that used VRelax the majority (53 of the 86, 62%) thought it was helpful to them (see Table 3).

**Table 3:** VRelax use: frequency and experiences of 86 ICU nurses.

Frequency of VRelax use, mean (SD)	2 (2)
Was VRelax easy to use?, *N* (%)	
No, not at all	1 (1%)
No	0 (0%)
No opinion	8 (9%)
Yes	49 (57%)
Yes, very easy	28 (33%)
Was it helpful to use VRelax?, *N* (%)	
No, not at all	4 (5%)
No, not really	11 (13%)
No opinion	18 (21%)
Yes	36 (42%)
Yes, very helpful	17 (20%)
Is it useful to have access to VRelax?	
On the wards, *N* (%)?	
Yes	59 (69%)
No	27 (31%)
At home?, *N* (%)	
Yes	25 (29%)
No	61 (71%)

Fifty-one ICU nurses (59% of VRelax users) commented on the open-ended question: “How did you experience using VRelax?” Answers were clustered whereby the following themes emerged: perceiving a pleasant getaway, quick effect, easy to use, and the feeling to be short on time for using VRelax.

Experiences: eleven nurses stated that they experienced immediate decrease of stress, another nine nurses mentioned they could leave daily hassles behind and experienced a sense of “a pleasant getaway” after the use of VRelax. One nurse remarked that her stress resilience was increased after the use of VRelax. Two ICU nurses reported that they experienced no effect of VRelax. Four nurses experienced negative physical sensations, such as nausea or dizziness.

*“The beautiful relaxing views give you the feeling of a pleasant getaway” 29, female.*

*“For a while, you are away from your daily work hassles” 26, female.*

Facilitating factors for the use of VRelax were the perceived effectiveness, the rapidity of the effect of VRelax, the guidance of the trained (para)medical students and easy-to-use interface of VRelax.

*“It [VRelax] was easy to work and gave quick effect” 48, female.*

One-third of the nurses stated that they felt too short on time, not having enough time during their work to use VRelax and/or could not assign their work to a colleague to take a break during work time due to high workload. The following quote is illustrative:

*“Due to the workload, I did not feel comfortable to assign my work to a colleague. When I was on a break, my colleague had to take care of 5 patients during that time.” 55, female.*

## Discussion

The aim of this study was to investigate feasibility and immediate effect of VRelax use on perceived stress among ICU nurses during the first wave of the COVID-19 pandemic. Our data showed that a quarter of all nurses used VR relaxation on demand during work shifts in the most intense 2months of the first COVID-19 wave. Immediate effect of VRelax on perceived stress was strong with a reduction of almost 40% (39.9%) in 10min. Most nurses enjoyed VRelax, found it easy to use, thought it was helpful and recommended regular availability of VRelax on the ICU wards. Main barrier for using VRelax was the experience that workload did not permit a 10-min break.

A unique quality of VR is the ability to provide a feeling of being completely present in another reality, leaving all “daily hassles” behind, leading to a pleasant getaway. The proposed components of action behind this effect is that the multisensory input (immersion) of virtual reality provides psychological “a sense of being there” (presence) evoking a physiological reaction (decreased stress response) corresponding to the calming valence of the environment (Gerber et al., 2017; Weech et al., 2019). This is in accordance with the previous literature describing that a “sense of presence” is correlated with an autonomic nervous system response in line with the valence of the VR environment (e.g., anxiety- or relaxation evoking; Gerber et al., 2017; Tsai et al., 2018). Our data underlines the idea that VR relaxation has the ability to enhance emotion-focused coping and meaning-focused coping strategies (Botella et al., 2013; Herrero et al., 2014). Depending on the situation (e.g., transient stressor or chronic stressor) VR relaxation can be part of emotion-based coping or either meaning-focused coping. VR relaxation can be used to introduce an immediate positive affective state, as part of emotion-based coping in the time of a transient stressor. When the stressor endures and becomes chronic VR relaxation can be part of meaning-focused coping, by supporting revalued goals (e.g., maintain mental well-being).

VRelax is an easy-to-use and stand-alone intervention. It can be used on demand; it is portable and therefore can be used whenever and wherever the user wants. Notable, VRelax was used by almost a quarter of the ICU nurses, despite the challenging times and uncontrollable circumstances they had to endure during the COVID-19 pandemic. Important barriers for use of VRelax was the experienced workload and inability to allot time for relaxation, which was in contrast with the fact that almost all respondents found stress resilience (very) important. This is a relevant finding, because sustainable employability of ICU nurses and other healthcare staff is needed for long-term perseverance of healthcare processes in these and other challenging times.

To our best knowledge is this the first paper investigating the effect of virtual reality on perceived stress in healthcare workers. A strength of this study is that we measured perceived stress using a visual analogue scale-stress (VAS-stress) scoring stress from 0 to 100. A single VAS-stress item is not as rich in describing perceived stress in comparison with other perceived stress scales such as the PSS-14 or State Trait Anxiety Inventory. We made a reasoned choice using scores on the VAS-stress as the mean measure as use and evaluation of VRelax on the ICU had to be convenient and as less time-consuming as possible. The single VAS-stress item facilitated this. Earlier research showed that a VAS-stress measuring perceived stress is considered a valid instrument and comparable with the 14-item perceived stress scale (Lesage and Berjot, 2011) in a linear association. Earlier research showed that a VAS-stress measuring perceived stress is considered a valid instrument and comparable with the 14-item perceived stress scale (Lesage and Berjot, 2011), in a linear association.

A limitation of this paper is the study design, no control group nor randomization of the intervention was performed, which limits the generalizability of the study. Most importantly, nonintervention-specific effects, such as taking time for yourself, sitting on a chair, or taking a break could have also induced relaxation, thereby overestimating the actual effect of VRelax. However, we do expect intervention-specific effects as these were shown previously in our recent randomized crossover (Veling et al., 2021). Taking into account that this trial has been done in a different population, namely a group of patients with a psychiatric diagnosis. As stress was measured directly before and after the use of VRelax, in a time span where the intensity of COVID-19 pandemic did not change, we do not expect that time effects have influenced the results (e.g., COVID-19 slowing down, thereby relieving experienced stress).

One case showed a reliable change when using the Reliable Change Index. No test–retest value for the VAS-stress was available; therefore, we used the most conservative r value available for VAS-anxiety, leading to a possible underestimating of the actual reliable change. The relatively large SD of the pretreatment stress scores suggests a higher heterogeneity within in the participants, possibly leading to an underestimation of the actual reliable change. For instance, 62% of the nurses who used VRelax stated in the questionnaire that VRelax was helpful.

Possibly, only nurses who felt enthusiastic about this type of intervention used VRelax. Some of the nurses commented that they experienced too much workload and therefore could not find the time to use VRelax. It is possible a selection bias is present in which the nurses with the highest level of perceived stress or the least interest in VRelax did not use VRelax. Although levels of stress were similar in VRelax users and non-VRelax users, an overestimation of the effect could be present which urges cautious interpretation of the results. It is also possible that just “a pleasant getaway” from work produced the reduction in perceived stress. However, what argues against this is that in a previous study we found that VRelax when compared to standard relaxation exercises produces a greater improvement in terms of positive mood states, such as cheerfulness (Veling et al., 2021). The additive effect of VR has been confirmed in a broad range of VR studies. For example, studies in the field of pain reduction show that immersive and interactive VR distraction techniques are more effective in reducing pain than non-immersive techniques (e.g., 2D videos or 2D gaming) by inducing upregulation of non-painful neurological signaling (Li et al., 2011, 2017).

This study took place during the COVID-19 pandemic, a time where healthcare workers, especially those working on an ICU, experience high levels of chronic stress. The results of this study support that short mental breaks using VRelax can be an effective intervention to reduce immediate perceived stress in ICU nurses providing COVID-19 care. VRelax was experienced as useful and easy to use by ICU nurses which makes VRelax a promising and feasible intervention for healthcare workers faced with the challenges during a pandemic or faced with other enduring stressful situations. A more rigorous study design (e.g., RCT or *N*=1 design) is needed to reduce potential bias and confirm stress-reducing effects and investigate longer-term effects on resilience, coping, and outcomes such as burnout and sick leave.

## Data Availability Statement

The raw data supporting the conclusions of this article will be made available by the authors, without undue reservation.

## Ethics Statement

The studies involving human participants were reviewed and approved by Medical Ethical Committee of the University Medical Centre Groningen (UMCG). The patients/participants provided their written informed consent to participate in this study.

## Author Contributions

JN contributed to the analyses, interpretation of the results, and writing the article. BL did the data collection, contributed to the design, analyses and interpretation of the results, and revision of the manuscript. WV contributed to the design, interpretation of the results, and revision of the manuscript. CD conceived the design, data collection, interpretation of the results, and writing and revision of the manuscript. All authors contributed to the article and approved the submitted version.

## Conflict of Interest

WV is cofounder and chief scientific officer of VRelax B.V., the company that has developed the VR in collaboration with UMCG, and holds shares in VRelax B.V. Statistical analyses has been done by JN and BL to prevent the impression of conflict of interest.

The remaining author declares that the research was conducted in the absence of any commercial or financial relationships that could be construed as a potential conflict of interest.

## Publisher’s Note

All claims expressed in this article are solely those of the authors and do not necessarily represent those of their affiliated organizations, or those of the publisher, the editors and the reviewers. Any product that may be evaluated in this article, or claim that may be made by its manufacturer, is not guaranteed or endorsed by the publisher.
